# Supplementary material for: Direct bandgap quantum wells in hexagonal Silicon Germanium
Source: Nat Commun. 2024 Jun 19;15:5252. doi: 10.1038/s41467-024-49399-3 (PMC11187182; doi:10.1038/s41467-024-49399-3)
Supplement: Supplementary file 1 — Supplementary Information [file 41467_2024_49399_MOESM1_ESM.pdf]

Direct bandgap quantum wells in hexagonal Silicon Germanium

Wouter H.J. Peeters,[1,*] Victor T. van Lange,[1,*] Abderrezak Belabbes,[2,3,*] Max C. van Hemert,[1]
Marvin Marco Jansen,[1] Riccardo Farina,[1] Marvin A.J. van Tilburg,[1] Marcel A. Verheijen,[1,4]
Silvana Botti,[3,5] Friedhelm Bechstedt,[3] Jos. E.M. Haverkort,[1] and Erik P.A.M. Bakkers[1,†]

[1] *Department of Applied Physics, Eindhoven University of Technology, 5600 MB Eindhoven, The Netherlands*
[2] *Department of Physics, Sultan Qaboos University, P.O. Box 123, Muscat, Oman*
[3] *Institut für Festkörpertheorie und -optik, Friedrich-Schiller-Universität Jena, Jena, Germany*
[4] *Eurofins Materials Science Netherlands BV, 5656 AE Eindhoven, The Netherlands*
[5] *Research Center Future Energy Materials and Systems of the University Alliance Ruhr and Interdisciplinary Centre for Advanced Materials Simulation, Ruhr University Bochum, Universitätsstraße 150, D-44801 Bochum, Germany*

I. SUPPLEMENTARY FIGURES

* These authors contributed equally to this work.
† e.p.a.m.bakkers@tue.nl

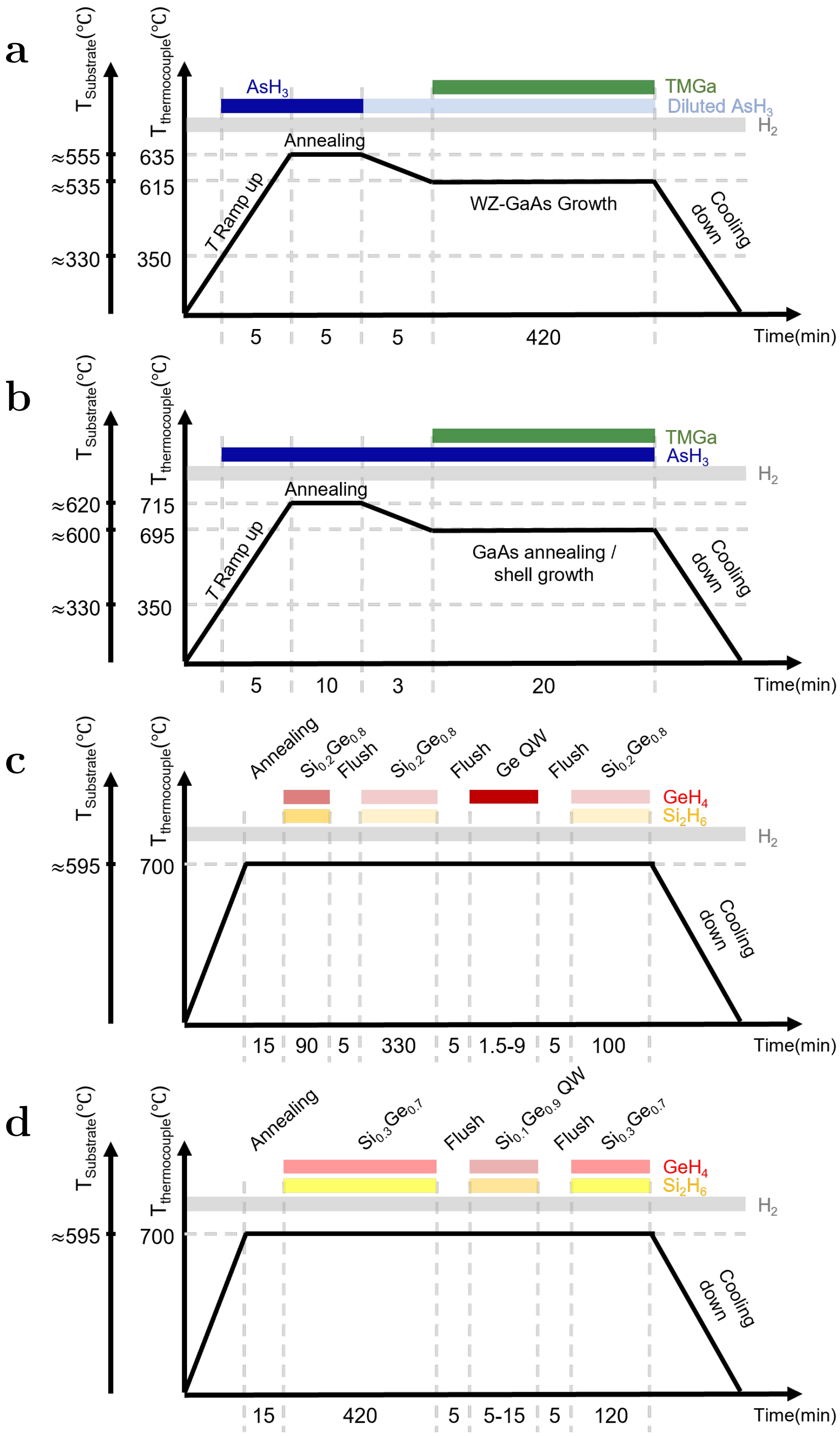

Figure S1: Recipes used to create QWs from $Si_{1-x}Ge_x$ alloys. a) Growth of WZ GaAs cores. Molar fractions of AsH_3, diluted AsH_3 and TMGa are $6.10 \cdot 10^{-3}$, $4.55 \cdot 10^{-5}$ and $1.91 \cdot 10^{-5}$ respectively. **b)** Cleaning of the WZ GaAs cores after Au removal. Molar fractions of AsH_3 and TMGa are $2.0 \cdot 10^{-3}$ and $1.52 \cdot 10^{-5}$ respectively. **c)** Recipe used for hex-Ge/$Si_{0.2}Ge_{0.8}$ QWs. The initial $Si_{0.2}Ge_{0.8}$ layer around the core is grown with GeH_4 and Si_2H_6 molar fractions of $7.26 \cdot 10^{-5}$ and $4.94 \cdot 10^{-6}$. The majority of the barrier is grown with molar fractions of $3.78 \cdot 10^{-5}$ and $2.12 \cdot 10^{-6}$. The Ge QW is grown for variable growth time at $1.71 \cdot 10^{-4}$. **d)** Recipe used for hex-$Si_{0.1}Ge_{0.9}$/$Si_{0.3}Ge_{0.7}$ QWs. The barrier is grown with GeH_4 and Si_2H_6 molar fractions of $3.54 \cdot 10^{-5}$ and $4.38 \cdot 10^{-6}$, while the QW is grown for variable growth time with $3.90 \cdot 10^{-5}$ and $1.06 \cdot 10^{-6}$ respectively.

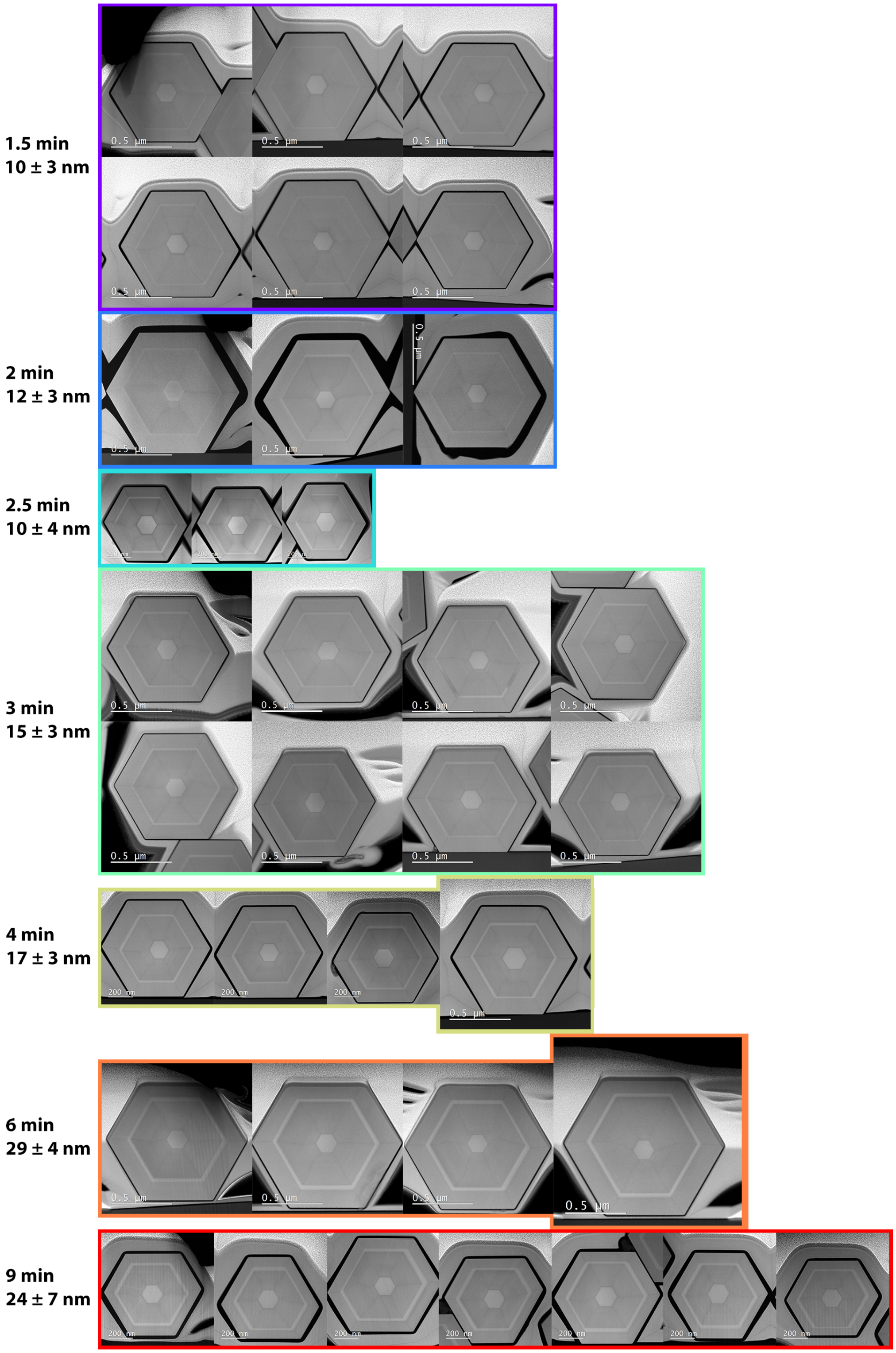

Figure S2: TEM study of Ge/$Si_{0.2}Ge_{0.8}$ QWs along the [0001] zone axis. Overview of High-Angle Annular Dark-Field Scanning Transmission Electron Microscopy (HAADF-STEM) images, scaled such that all images have the same magnification. The GaAs core has approximately the same size in all NWs. The total diameter of the NW however fluctuates between samples, mainly due to a varying $Si_{0.2}Ge_{0.8}$ growth rate. Higher resolution images are used to measure the Ge QW thickness on each of the six facets of the NW.

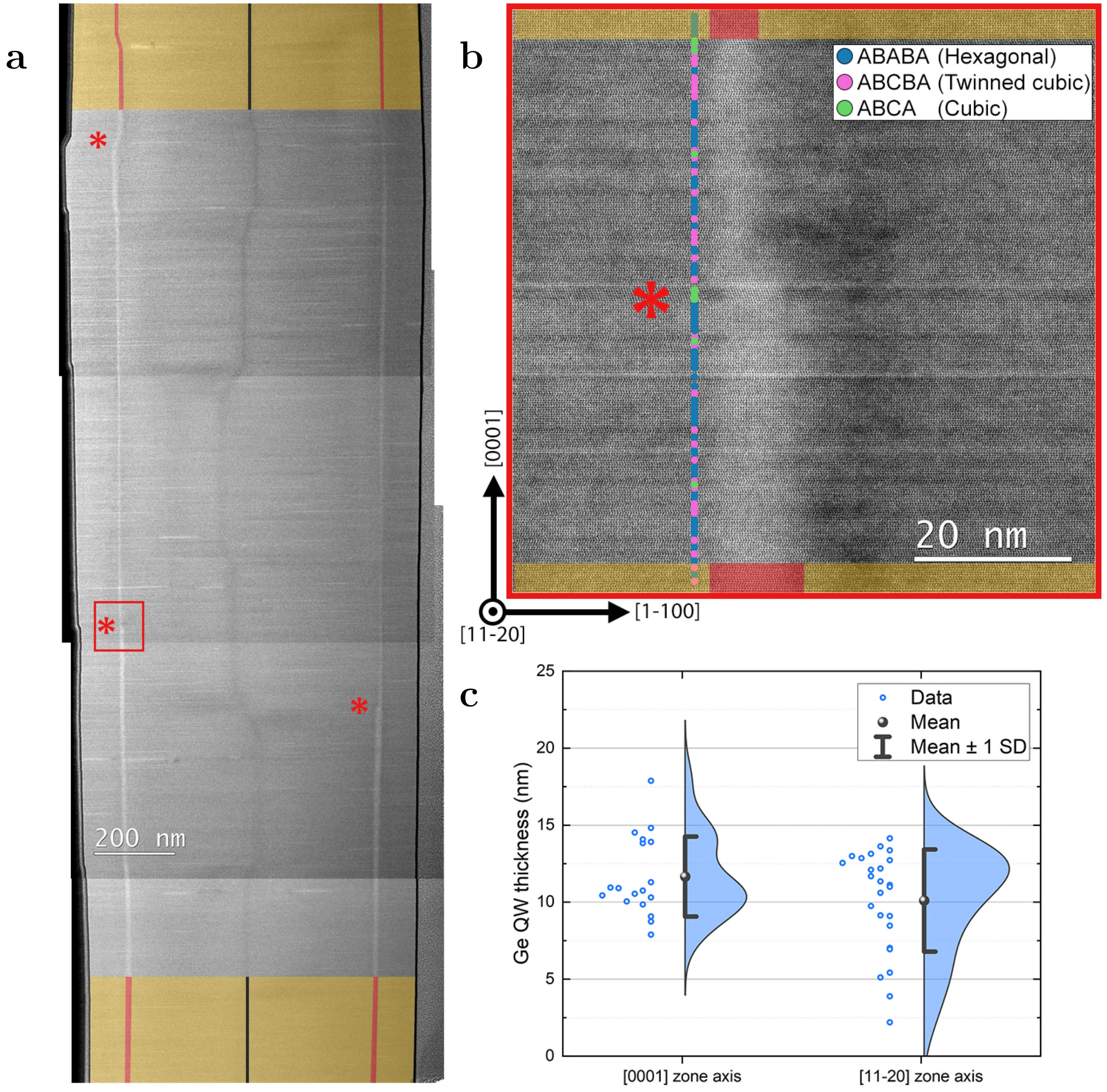

Figure S3: TEM study of a Ge/$Si_{0.2}Ge_{0.8}$ QW along the [$11\bar{2}0$] zone axis. a) Stitched HAADF-STEM images of a single NW with a (12 ± 3) nm (2.0 min) Ge/$Si_{0.2}Ge_{0.8}$ QW. The Ge QW is visible as two vertical lines (partly indicated with the red shading). The dark line in the center of the NW is the Si-rich spoke, the GaAs core is not included in this lamella. The Ge QW changes thickness and position, as indicated by the red asterisks *. Such region is more closely studied in **b)**, where locations with local hexagonal (ABABA, blue), cubic (ABCA, green), and twinned cubic boundary (ABCBA, pink) stacking are indicated with circles. The abrupt change in QW thickness is near an elongated cubic segment of 7 monolayers. **c)** Ge QW thickness, measured on multiple NWs imaged along the [0001] zone axis, compared to the QW thickness measured on a single NW imaged along the [$11\bar{2}0$] zone axis. Relatively more small thicknesses are found in the [$11\bar{2}0$] zone axis. This is likely because the thicknesses are imaged in transmission, and therefore a superposition of local QW thicknesses within the thickness of the TEM lamella is measured. To measure a small thickness along the [0001] zone axis, the QW throughout the entire lamella should be of the same thickness, and this is unlikely since the QW thickness on a single facet varies more along the [0001] than the [$11\bar{2}0$] direction. Thus, the average thicknesses presented in Fig. 2b and 3b of the main text are slightly overestimating the true average QW thickness, but the thicknesses in both zone axes overlap when considering the standard deviation.

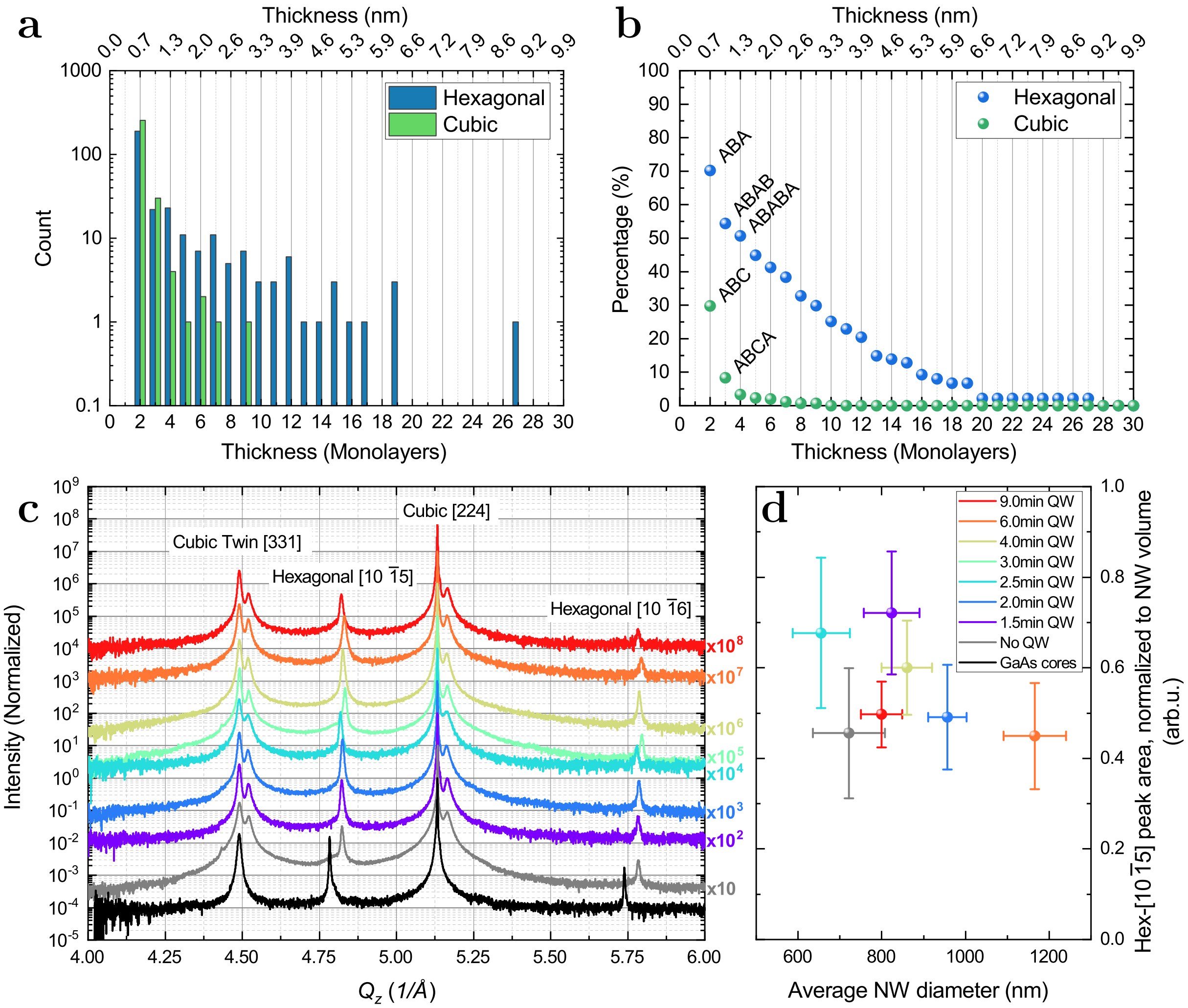

Figure S4: Crystal structure in the Ge/$Si_{0.2}Ge_{0.8}$ QWs. a) Statistical analysis of the atomic stacking sequence in the (12 ± 3) nm (2.0 min) Ge/$Si_{0.2}Ge_{0.8}$QW. The stacking is counted in the number of monolayers, defining alternating ABA, ABAB, and ABABA as being 2, 3, and 4 monolayers of hexagonal stacking respectively. On the other hand, ABC, ABCA, and ABCAB are counted as 2, 3, and 4 monolayers of coherent cubic stacking respectively. The length of hexagonal stacking has a broad distribution, and the maximum length of hexagonal stacking observed is 27 monolayers. In contrast, coherent cubic sections without twinning defects are less frequent and shorter in length. The histogram is obtained by studying several positions of the lamella displayed in Fig. S3. The total length studied for this analysis is approximately 400 nm. **b)** Weighted sum of segment lengths, displaying what percentage of the NW has a certain stacking sequence. For example, around 70% (50%) of the NW belongs to a hexagonal segment that is *at least* 2 (4) monolayers ABA (ABABA) long (See main manuscript, Methods). The minimum hexagonal segment length, which still has a direct bandgap and thus contributes to light emission, is unknown and subject to future research. **c)** Line scan of the out-of-plane reciprocal lattice coordinate Q_z through the asymmetric $[10\bar{1}L]$ crystal truncation rod. Offsets are applied for better visibility. The scan includes the cubic [224] reflection, which mainly originates from the GaAs substrate. Other peaks, such as the hexagonal $[10\bar{1}5]$, $[10\bar{1}6]$, and cubic twin [331] appear only after the growth of the NWs. A sample with only WZ GaAs cores shows the presence of the hexagonal reflections, but also a significant peak from a cubic crystal with twinned orientation relative to the substrate. Growth of a $Si_{0.2}Ge_{0.8}$ shell shifts the hexagonal reflection to higher Q_z (i.e. lower out-of-plane lattice constant). In contrast, the twinned cubic reflection does not shift significantly. Instead, both the cubic and twinned cubic reflections get an additional shoulder at higher Q_z. The shoulder is likely related to cubic $Si_{0.2}Ge_{0.8}$ growth on the top [0001] facet of the NWs, which can have both twin orientations, but contributions from the cubic defects in the shell are not ruled out. **d)** The amount of hexagonal crystal stacking within each sample is compared by evaluating the area under the $[10\bar{1}5]$ peak, and normalizing it for the volume of the NWs (See main manuscript, Methods). Samples with larger NW diameters have slightly less hexagonal stacking in them, but the relative hexagonal XRD intensities are similar, indicating comparable crystal quality between samples. Horizontal error bars indicate the standard deviation of NW diameter as determined from SEM images, which are also used to calculate the vertical error bars, in combination with the uncertainty of XRD peak area.

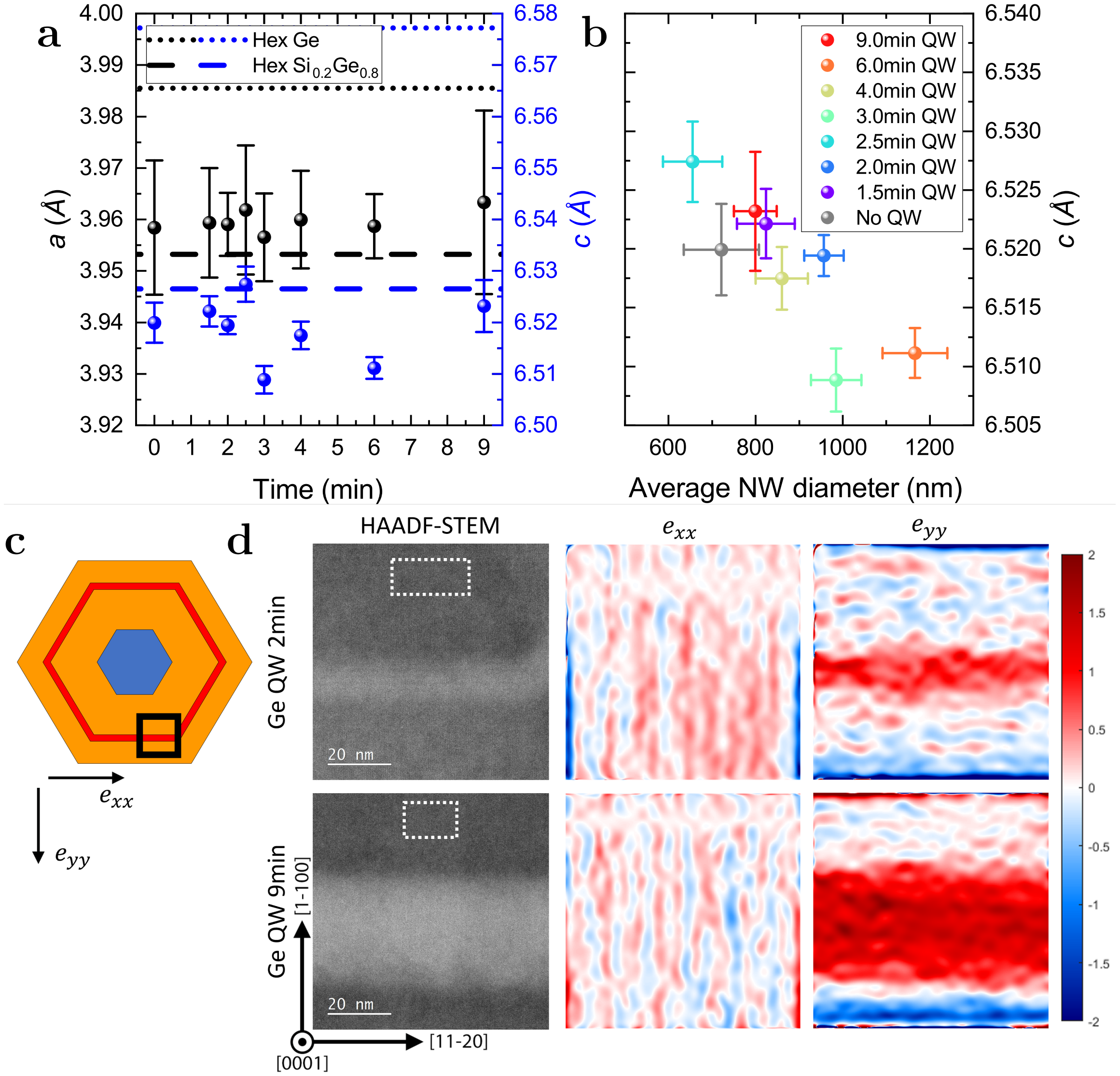

Figure S5: Lattice constants of the QWs. a) Average lattice parameters of the $GaAs/Si_{0.2}Ge_{0.8}/Ge/Si_{0.2}Ge_{0.8}$ NWs, for samples with a different Ge growth time as obtained by X-ray diffraction measurements of an ensemble of NWs. Reference lattice constants of hex-Ge and hex-$Si_{0.2}Ge_{0.8}$ are included as obtained from Vegard's law are included as horizontal lines [1]. Out-of-plane lattice constants c are more accurate than the in-plane lattice constants a due to small tilts of individual NWs relative to the substrate. All samples have comparable lattice constants, independent of the QW thickness. The largest strain difference between samples is 0.28 %, relative to relaxed hex-Ge. The datapoints are derived from the peak position of the hexagonal $[10\bar{1}5]$ reflection (Fig. 2d of the main text), error bars correspond to the uncertainty of the peak position. **b)** The different lattice constants in each sample are related to the total NW diameter, which is mainly determined by the $Si_{0.2}Ge_{0.8}$ shell thicknesses (Fig. S2). **c)** Schematic cross-section of the NW, highlighting the two strain directions e_{xx} and e_{yy}. The black square is the region of the NWs used for Geometric Phase Analysis (GPA) shown in panel **d)** Local lattice constants in the $Ge/Si_{0.2}Ge_{0.8}$ QW, as obtained by GPA of HAADF-STEM images. Left panels show two QWs along the [0001] zone axis. These Atomic-resolution HAADF-STEM images of different QW thicknesses are used to calculate the local variations of the lattice parameter for the e_{xx} (azimuthal) and e_{yy} (radial) directions, using the regions in the dashed rectangle as a reference of net-zero strain. For both samples, the e_{xx} maps show noise around zero, while the e_{yy} maps show a clear positive value in the QW region. From the e_{xx} maps, we conclude that the lattice constants in the azimuthal direction of the barrier and QW are equal, implying that the Ge is compressed in the azimuthal direction. Due to this compression, the radial lattice constant of the Ge increases, as measured in the e_{yy} maps. This effect is similar to the Poisson effect. The magnitude of the radial expansion is related to the QW thickness. This situation resembles pseudomorphic growth of Ge on the lattice-mismatched $Si_{0.2}Ge_{0.8}$.

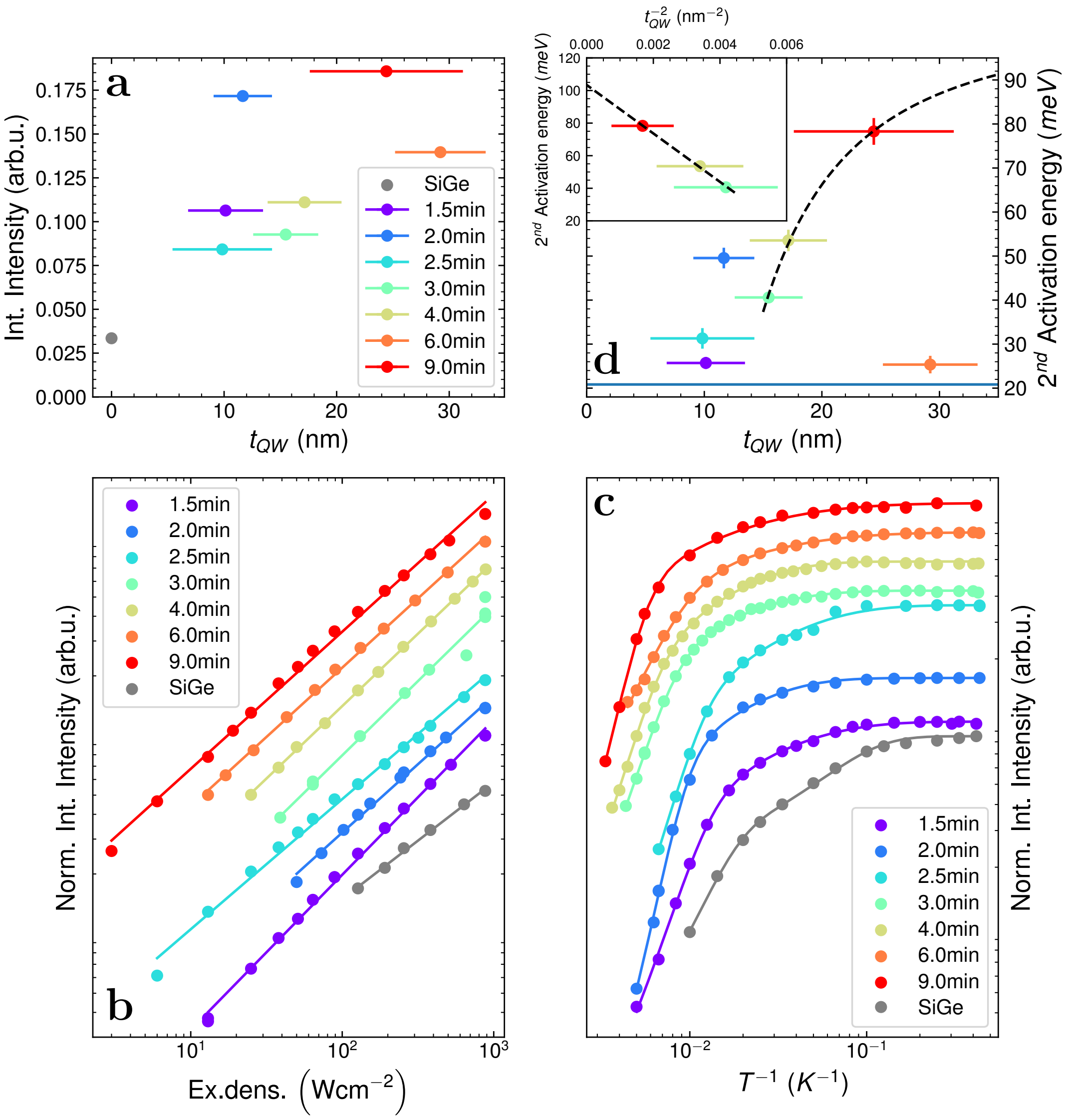

Figure S6: Comparison of the photoluminescence from different Ge/Si$_{0.2}$Ge$_{0.8}$ QWs. a) The integrated PL intensity versus the measured QW width for the different QWs compared to the bulk hex-Si$_{0.2}$Ge$_{0.8}$ barrier intensity, placed at 0 nm in grey, measured at T =4 K and P =0.88 kW cm^{-2} in as-grown configuration. The total PL intensity of the QW samples is on average 3.8x (between 2.5x and 5.5x) higher than the barrier reference sample. Error bars in t_{QW} are the standard deviations presented in Fig. 2b of the main text. **b)** Light-In Light-Out (LILO) curves at T =4 K and **c)** Modified Arrhenius[2] plots at P =0.88 kW cm^{-2} of the different QWs and the hex-Si$_{0.2}$Ge$_{0.8}$ barrier reference. All QWs outperform the Si$_{0.2}$Ge$_{0.8}$ reference in thermal stability of the emission intensity. This is expected as the confined carriers are shielded from non-radiative trap states in the bulk. Additionally, it is observed that for wider QWs, (higher ionization energy), the samples emit strong up to higher temperatures. The LILO curves show a slope of 0.59 ± 0.02 for the bulk Si$_{0.2}$Ge$_{0.8}$ reference sample while showing slopes $0.69 - 0.78$ for the QW samples. This also shows the QWs are less sensitive to the non-radiative processes in the NW. From the Arrhenius fits in c), we investigate the second activation energy in **d)** which generally correspond to the thermal emission of the most shallow confined charge carrier[2, 3]. We observe a downwards trend towards smaller QW widths t_{QW}. For the three thickest quantum wells of comparable geometry (24, 17 and 15 nm) we can reasonably assume that they behave approximately as infinite quantum wells which allows us to estimate the band-offset and effective mass of the one of the charge carriers by fitting with a linear relation shown in the inset. We find a $E_{offset} = (100 \pm 30)$ meV and $m^* = (0.03 \pm 0.02)\, m_0$ which correlates well with the theoretical prediction and shows that the band offset distribution should be approximately equal. The theory is discussed further in the main text. The horizontal line at ≈20 meV shows the second activation energy of the hex-Si$_{0.2}$Ge$_{0.8}$ barrier reference. Error bars in the activation energy are the standard deviations obtained from the Arrhenius fits. All samples are normalized to their respective lowest temperature and highest power measurement and offset for clarity which correspond to the points in (a).

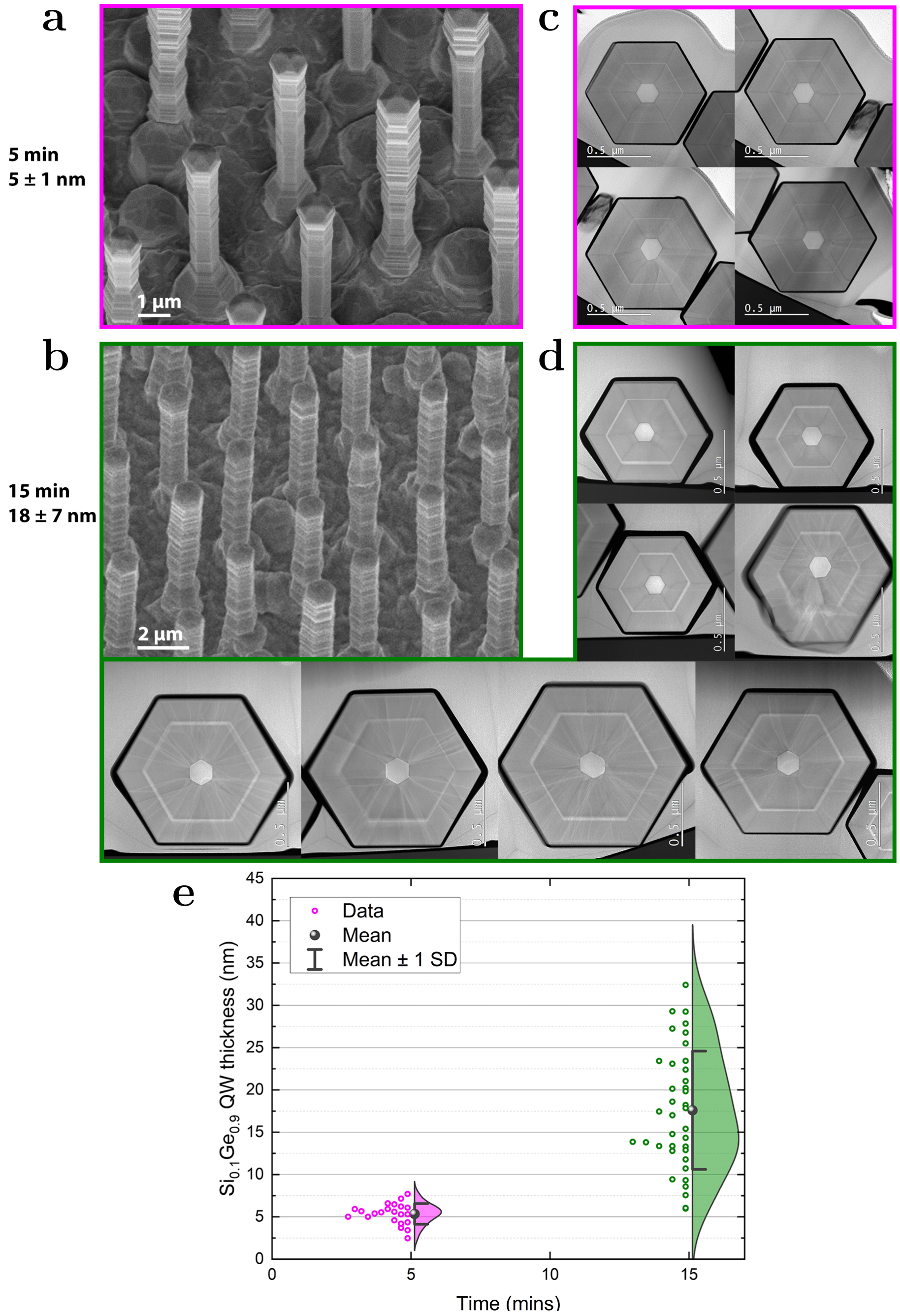

Figure S7: TEM study of the $Si_{0.1}Ge_{0.9}$/$Si_{0.3}Ge_{0.7}$ QW samples. 30-degree tilted Scanning Electron Micrograph (SEM) of NW arrays from **a)** the (5 ± 1) nm (5 min) and **b)** (18 ± 7) nm (15 min) samples respectively. On average, the NWs have less vertical side facets compared to Fig. 1c of the main text. This is indicative that the NWs have relatively more cubic stacking, likely due to the larger lattice mismatch between the GaAs core and $Si_{0.3}Ge_{0.7}$ compared to $Si_{0.2}Ge_{0.8}$ [1]. The 15 min sample was rotated differently in the SEM, hence the facets look rotated. **c-d)** Overview of all $Si_{0.1}Ge_{0.9}$/$Si_{0.3}Ge_{0.7}$ cross-sectional TEM lamellas imaged along the [0001] zone axis. The HAADF-STEM images are scaled such that all images have the same magnification. From the 15 min sample, 4 out of 8 have a GaAs core with rotated $\{11\bar{2}0\}$ facets instead of $\{1\bar{1}00\}$ facets. These GaAs cores are likely dominated by Zinc-Blende stacking, rather than Wurtzite. In these NWs, many dislocations can be identified as contrast lines in both core and shells. These dislocations also appear in the lamellas with non-rotated GaAs cores, but less numerous. **e)** Growth rate curve for $Si_{0.1}Ge_{0.9}$/$Si_{0.3}Ge_{0.7}$ QWs. The thicknesses of individual facets, all measured in images acquired along the [0001] zone axis, are indicated with the coloured data points. QWs of which the thickness could not be measured accurately, due to varying QW position or width within the thickness of the TEM lamella, are excluded from the analysis. Coloured areas show approximate probability distributions, obtained from these data points by Kernel smoothing. The average is taken as a measure of the QW thickness, which is taken as input for the finite QW model in Fig. 5c in the main text.

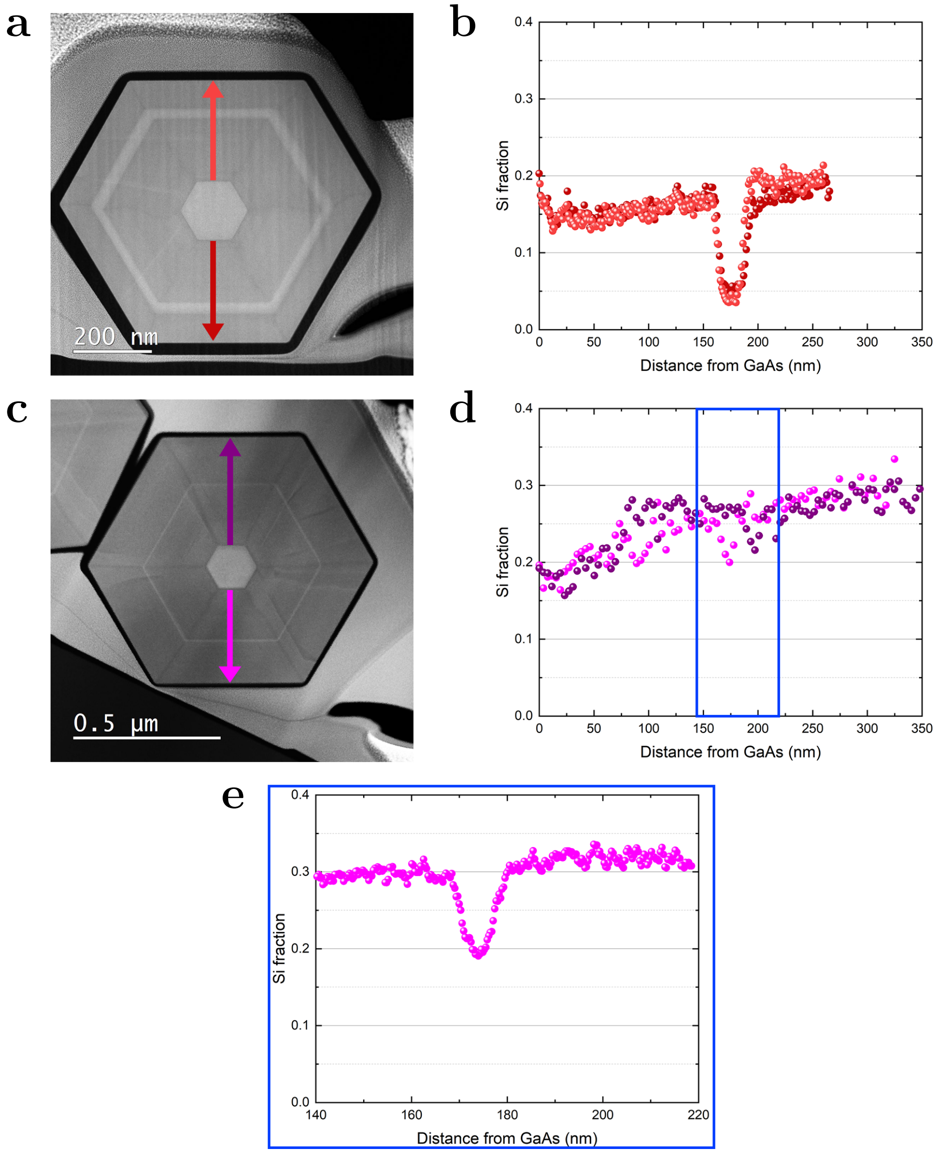

Figure S8: Energy-dispersive X-ray (EDS) spectroscopy study of QWs. a) Cross-sectional lamella of the (24 ± 7) nm (9 min) Ge/$Si_{0.2}Ge_{0.8}$ QW sample. **b)** Radial Si fraction profile extracted from the quantified EDS mapping, averaged over a width in the image identical to the GaAs facet length. The Si fraction in the QW drops to 5 %, which is an over-estimation (of approximately 2 %) from stray radiation coming from the Si substrate on which the lamella is made. **c)** Cross-sectional lamella of the (5 ± 1) nm (5 min) $Si_{0.1}Ge_{0.9}$/$Si_{0.3}Ge_{0.7}$ QW. **d)** Radial Si fraction profile extracted from the quantified EDS mapping. The Si fraction increases throughout the shell. **e)** High-magnification scan of the Si fraction near the $Si_{0.1}Ge_{0.9}$ QW. The Si concentration measured by EDS in the QW is going down to 20 %. This value may be an overestimation of the Si content in the QW, as it may be affected by the pixel size of the mapping as well as by broadening of the incident electron beam within the thickness of the TEM lamella. The Si concentration in the barrier around the QW is approximately constant.

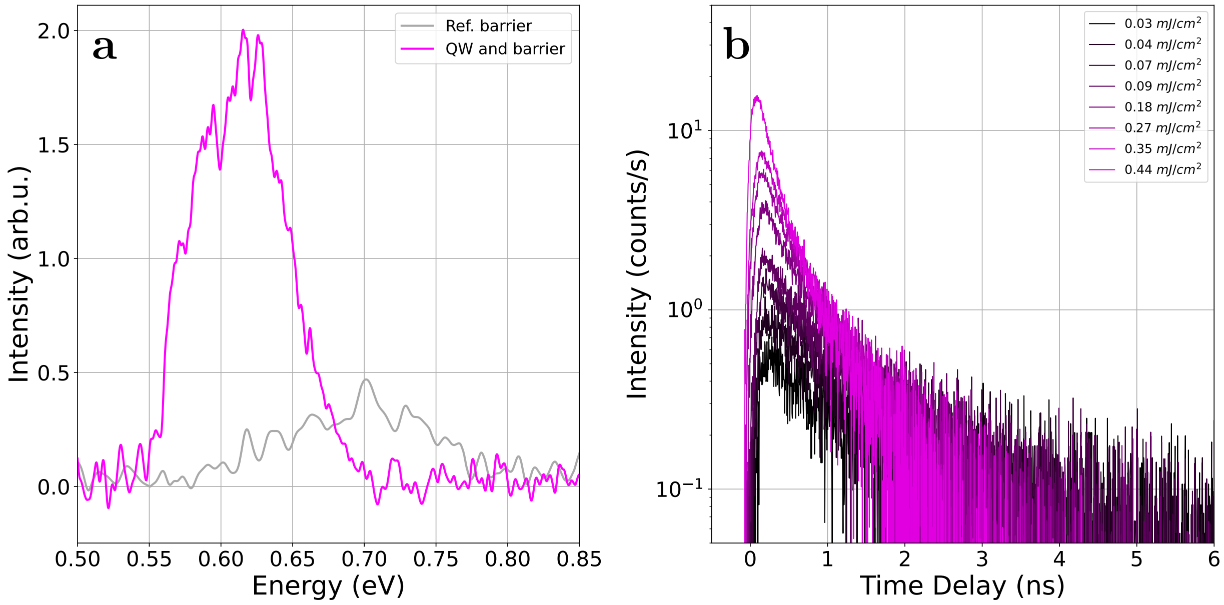

Figure S9: Micro-photoluminescence study of single NWs with a $Si_{0.1}Ge_{0.9}/Si_{0.3}Ge_{0.7}$ QW. a) Normalized emission spectrum of a single nanowire swiped on an AlN planar substrate. The magenta line represents the spectrum from the thinnest QW, while the grey line is the barrier reference. Excitation densities for the QW and barrier reference are $0.09\,\mathrm{mJ\,cm^{-2}}$ and $0.88\,\mathrm{mJ\,cm^{-2}}$ respectively showing a significantly brighter emission from the QW. **b)** Time decay of the integrated photoluminescence intensity from $Si_{0.1}Ge_{0.9}/Si_{0.3}Ge_{0.7}$ QW for increasing laser fluence. The measurement is performed on a single nanowire swiped on an AlN substrate. The excitation densities are ranging from $27\,\mathrm{\mu J\,cm^{-2}}$ (black) to $442\,\mathrm{\mu J\,cm^{-2}}$ (magenta). At the lowest excitation density, we observe an almost mono-exponential decay with a lifetime of (1.3 ± 0.2) ns which we attribute to "mono-molecular" radiative recombination inside the QW showing a similar lifetime as reported by Fadaly et. al. [1].

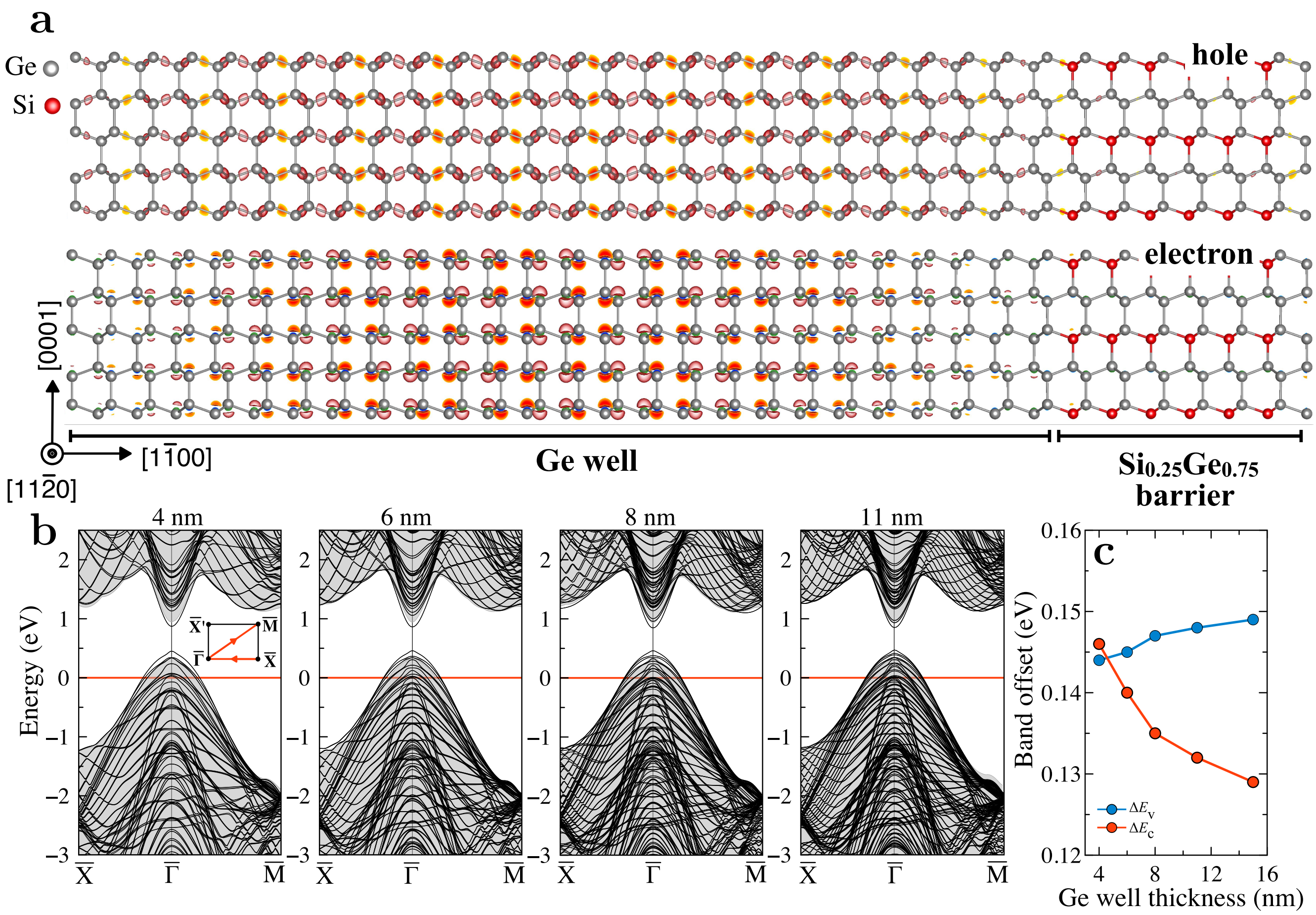

Figure S10: *Ab initio* simulations of hex-Ge/$Si_{0.25}Ge_{0.75}$ heterostructures. a) Square of the wave function of the lowest electron and highest hole subband at $\bar{\Gamma}$ of the 8 nm QW structure. Both wave functions are localized in the hex-Ge layer, indicating type-I behaviour. **b)** Band structures of hex-Ge/$Si_{0.25}Ge_{0.75}$ MQW structures with almost constant barrier thickness of t_{SiGe} =2 nm but varying hex-Ge QW thickness of about t_{Ge} =4 (left panel), 6, 8 and 11 nm (right panel) with external strain. The grey background illustrates the projected bulk band structure of the correspondingly biaxially strained $Si_{0.25}Ge_{0.75}$. The MQW band structure and the projected bulk band structure are aligned using the Branch Point energies. The BP is used as energy zero in all panels. Consequently, the conduction and valence bands around $\bar{\Gamma}$ in the fundamental gap but embedded by the projected bulk conduction and valence bands represent electron or hole subbands with wave functions localized in the hex-Ge QWs. **c)** Band offsets of the Ge/$Si_{0.25}Ge_{0.75}$ heterostructures that characterize the depths of the corresponding finite rectangular well potentials ruling the quantization and localization of electrons and holes in the Ge wells. They are derived from the conduction and valence band extrema at $\bar{\Gamma}$ of the bulk Ge well and the $Si_{0.25}Ge_{0.75}$ barrier materials.

[1] Fadaly, E. M. T. *et al.* Direct-bandgap emission from hexagonal Ge and SiGe alloys. *Nature* **580**, 205–209 (2020).
[2] Fang, Y. *et al.* Investigation of temperature-dependent photoluminescence in multi-quantum wells. *Scientific Reports* **5**, 12718 (2015).
[3] Gurioli, M. *et al.* Thermal escape of carriers out of GaAs/Al x Ga 1- x As quantum-well structures. *Physical Review B* **46**, 6922 (1992).
